# Adipocyte-induced transdifferentiation of osteoblasts and its potential role in age-related bone loss

**DOI:** 10.1371/journal.pone.0245014

**Published:** 2021-01-26

**Authors:** Aline Clabaut, Céline Grare, Gaëlle Rolland-Valognes, Jean-Guillaume Letarouilly, Chantal Bourrier, Thomas L. Andersen, Tanja Sikjær, Lars Rejnmark, Charlotte Ejersted, Philippe Pastoureau, Pierre Hardouin, Massimo Sabatini, Odile Broux

**Affiliations:** 1 Marrow Adiposity and Bone Lab, MABLab ULR4490, Univ. Littoral Côte d’Opale, Boulogne-sur-Mer, France; 2 Univ. Lille, Lille, France; 3 CHU Lille, Lille, France; 4 CentExBiotechnology, Servier Research Institute, Croissy-sur-Seine, France; 5 Department of Pathology, Odense University Hospital, Odense, Denmark; 6 Department of Clinical Research & Department Molecular Medicine, University of Southern Denmark, Odense, Denmark; 7 Department of Forensic Medicine, Aarhus University, Aarhus, Denmark; 8 Department of Endocrinology and Internal Medicine, Aarhus University Hospital, Aarhus, Denmark; 9 Department of Endocrinology, Odense University Hospital, Odense, Denmark; 10 CTI Immuno-Inflammatory Disease, Servier Research Institute, Croissy-sur-Seine, France; Università degli Studi della Campania, ITALY

## Abstract

Our preliminary findings have lead us to propose bone marrow adipocyte secretions as new contributors to bone loss. Indeed, using a coculture model based on human bone marrow stromal cells, we previously showed that soluble factors secreted by adipocytes induced the conversion of osteoblasts towards an adipocyte-like phenotype. In this study, microarray gene expression profiling showed profound transcriptomic changes in osteoblasts following coculture and confirmed the enrichment of the adipocyte gene signature. Double immunofluorescence microscopic analyses demonstrated the coexpression of adipogenic and osteoblastic specific markers in individual cells, providing evidence for a transdifferentiation event. At the molecular level, this conversion was associated with upregulated expression levels of reprogramming genes and a decrease in the DNA methylation level. In line with these *in vitro* results, preliminary immunohistochemical analysis of bone sections revealed adipogenic marker expression in osteoblasts from elderly subjects. Altogether, these data suggest that osteoblast transdifferentiation could contribute to decreased bone mass upon ageing.

## Introduction

Bone marrow stromal cells (BMSCs) are progenitor cells that can self-renew and have multipotent differentiation potential and the ability to home into tissues, thus possessing great potential for the repair and regeneration of damaged tissues, such as cartilage and bone [1–4]. BMSCs can differentiate into various cell types, including adipocytes, osteoblasts and chondrocytes, which are all present in bone marrow and play crucial roles in bone metabolism [5, 6]. Maintenance of bone mass requires a continuous process of bone renewal, known as bone remodelling, which is regulated by the balance between bone resorption by osteoclasts and bone formation by osteoblasts. The decrease in bone mass that occurs in osteoporosis results from the impairment of this balance, leading to increased fracture risk. The strong relationship between this bone loss and the increase in bone marrow adipose tissue suggests a potential role for medullary adipocytes in the deficiency of osteoblasts to replace resorbed bone [7–9].

It has been suggested that the excessive accumulation of marrow adipocytes could be due to the preferential differentiation of BMSCs into adipocytes at the expense of osteoblasts or to the transdifferentiation of osteoblasts into adipocytes [10]. Transdifferentiation, also known as lineage reprogramming, is the conversion of one mature somatic cell into another mature somatic cell without going through a pluripotent state [11]. Several *in vitro* studies using human BMSCs or mature osteoblasts derived from trabecular bone have demonstrated the occurrence of transdifferentiation between osteoblastic and adipocytic phenotypes in response to extrinsic factors [11–14].

It is also conceivable that the osteoporosis-associated increase in marrow adipogenesis may contribute to limiting osteoblast commitment by acting on BMSCs or even directly on osteoblasts [15]. This hypothesis is supported by the proximity of adipocytes to osteoblasts in the bone marrow and the knowledge that adipocytes exert paracrine effects on neighbouring cells. Indeed, several *in vitro* studies have shown that adipocyte-secreted factors, such as adipokines or fatty acids, may modify the proliferation and function of osteoblasts [16–19].

In this context, determining the exact contribution of bone marrow adipocytes could be useful in the therapeutic control of bone loss. In an attempt to reproduce cellular interactions within the bone marrow, we previously developed a coculture system using human BMSC-derived osteoblasts and adipocytes. In this model, we demonstrated that soluble factors secreted by adipocytes induced the conversion of osteoblasts towards an adipocyte-like phenotype, as evidenced by the expression of adipogenic mRNA markers and the decrease in the levels of osteogenic mRNA markers [20].

In the present study, our aim was to analyse this conversion in more detail. Using temporal gene expression analysis, we showed that transcriptomic changes and adipogenic gene induction in osteoblasts were apparent as early as 9 hours of coculture. Using immunofluorescence microscopic analysis, we further demonstrated the coexpression of adipogenic and osteogenic proteins in cocultured osteoblasts, providing evidence for a transdifferentiation process. Interestingly, we found that this osteoblast transdifferentiation was accompanied by an increased expression level of reprogramming genes and a decrease in the global DNA methylation level, which suggest global epigenetic remodelling. Finally, to evaluate the role of this transdifferentiation event in age-related bone loss, we performed immunohistochemistry on human bone biopsy specimens, which revealed adipogenic marker expression in mature osteoblasts from elderly patients.

## Experimental procedures

### Cell culture experiments

#### Cell culture and induction of osteogenic and adipogenic differentiation

Purified commercial hBMSCs (Lonza) from 7 donors (4 females, A: 33 years, B: 22 years, C: 24 years, G: 23 years and 3 males, D: 19 years, E: 22 years, F: 25 years) were cultured as described previously [20]. Briefly, differentiation experiments were started when hBMSCs at passage 4 or 6 had reached confluence (D0). To induce osteogenesis, hBMSCs were cultured in DMEM with 10% FCS supplemented with osteogenic inductors (50 μM ascorbic acid, 10 mM β-glycerophosphate and 10−8 M vitamin D3 (Sigma-Aldrich)) for 14 days. For adipogenic differentiation, hBMSCs were cultured in DMEM with 10% FCS supplemented with adipogenic inductors (0.5 μM dexamethasone, 0.5 mM isobutyl-1-methylxanthine and 50 μM indomethacin (Sigma-Aldrich)) for 14 days.

#### Adipocyte and osteoblast coculture

hBMSCs from the same donor were either seeded on cell culture inserts (pore size of 0.4 μm, Millicell) at a density of 9 × 10^3^ cells per cm^2^ and cultured after confluence in adipogenic medium or plated in the basal compartment of 6-well plates at a density of 9 × 10^3^ cells per cm^2^ and cultured after confluence in osteogenic medium. After 14 days of induction, inserts were then transferred to 6-well plates, and cocultures (OB-CC) were maintained in serum-free DMEM for 9 h, 48 h or 72 h. Control osteoblasts (OB) were cultured with serum-free DMEM for the same time.

#### Conditioned medium experiments

On day 12 of differentiation, hBMSC-derived adipocytes (AD) were placed in serum-free DMEM for 48 h. The supernatants were then collected and applied immediately to hBMSC-derived osteoblasts at day 14 of differentiation. Osteoblasts were incubated in conditioned medium for 48 h (OB-CM). As controls, osteoblasts were incubated with serum-free DMEM for the same time (OB).

#### Oil Red O staining

Cells were fixed in 2% paraformaldehyde for 15 min, washed in water, incubated with 60% isopropanol for 5 min and stained with newly filtered Oil Red O solution for 10 min at room temperature. After staining, the cells were rinsed with water before counterstaining with Mayers-Haematoxylin for 5 min at room temperature.

### RNA expression measurement

#### RNA isolation

Total RNA was extracted using the RNeasy® Micro Kit, including the DNase I digestion step (Qiagen), according to the manufacturer’s instructions and quantified by a Nanodrop at the wavelength of 260 nm.

#### mRNA expression analysis

Total RNA was reverse transcribed using the Maxima First-Strand cDNA Synthesis kit (Thermo Scientific) and subjected to quantitative real-time PCR on the StepOnePlus® system (Applied Biosystem) using POWER SYBR® Green PCR Master Mix (Thermo Fisher) and specific primers designed using Oligo 6 software (MedProbe) (Table 1). Relative gene expression levels were normalized to YWHAZ (tyrosine 3-monooxygenase/tryptophan 5-monooxygenase activation protein), PPIA (peptidylprolyl isomerase A) and RP2 (RNA polymerase II) transcripts and determined using the 2 –ΔΔCt method. For the statistical analysis, differences between the control and OB-CC groups were compared using the Mann-Whitney test. A *p*-value of <0.05 was set as statistically significant.

**Table 1:** Primers used for RT-PCR analysis.

Gene	Primer Sequence (5' to 3')	Annealing temperature
PPIA	Forward; ACCGTGTTCTTCGACATTGC	55°C
	Reverse; CAGGACCCGTATGCTTTAGGA	
YWHAZ	Forward; GGT CAT CTT GGA GGG TCG TC	55°C
	Reverse; GTC ATC ACC AGC GGC AAC	
Tet1	Forward; CCCTTACCGGAGTCA	49,1°C
	Reverse; AAAGAAGTTCATGGCATAAT	
Oct-4	Forward; ATTCAGCCAAACGACCATC	60°C
	Reverse; GTTGCCTCTCACTCGGTTCT	
c-Myc	Forward; GCT GCC AAG AGG GTC A	55°C
	Reverse; CGC ACA AGA GTT CCG TAG	
PPARG	Forward; GCTTCTGGATTTCACTATGG	52°C
	Reverse; AAACCTGATGGCATTATGAG	
HSD11B1	Forward; CAGCAAAGGGATCGGAAGA	56,1°C
	Reverse; AAATTGCTCTGCGAAGGTCA	
RP2	Forward; CCAAGCAGGACGTAATAGAGG	55°C
	Reverse; CCGGACACGACCATAGACT	

### Microarray assays

#### Gene expression profiling

Total RNA from cocultured osteoblasts and control samples (9 h and 48 h, each n = 7 donors, A-G) was used for whole genome array analysis. RNA quality was confirmed using an Agilent 2100 Bioanalyser (Agilent Technologies). Gene expression data were produced using the Agilent Human SurePrint G3 Microarray, 8×60K microarray chip (Agilent Technologies). Microarray data preprocessing and statistical analysis were performed using the Limma R/Bioconductor package in R software and were filtered based on the FDR method of Benjamini – Hochberg and the fold-change. Only genes that were significantly (adjusted *p*-value of <0.05 and fold-change of >1.3) modulated were considered for further analysis. Microarray data have been deposited in the ArrayExpress database at EMBL-EBI (https://www.ebi.ac.uk/arrayexpress/experiments/E-MTAB-8849/).

Differentially expressed genes were analysed for gene set enrichment using the online version of GeneCodis3 (http://genecodis.cnb.csic.es) [21–23] and DAVID Bioinformatics Resources 6.8 (http://david.abcc.ncifcrf.gov) [24, 25].

#### Quantitative RT-PCR validation

Transcriptomic results were further validated using RNA from two donors (C and F). cDNA was synthetized as described above. Quantitative RT-PCRs were performed in duplicate on a StepOnePlus® system using TaqMan Universal Master Mix and selected TaqMan probes (Life Technologies) and using POWER SYBR® Green PCR Master Mix (Thermo Fisher) and specific primers designed using Oligo 6 software (MedProbe) (Table 2). The relative gene expression levels were normalized to YWHAZ (tyrosine 3-monooxygenase/tryptophan 5-monooxygenase activation protein) and PPIA (peptidylprolyl isomerase A) transcripts and determined by the 2 –ΔΔCt method.

**Table 2:** Probes used for RT-PCR analysis.

Gene ID	Taqman probes
ADM	Hs00969450_g1
ARID5B	Hs0138278_m1
CD36	Hs00354519_m1
CEBPA	Hs00269972 s1
DLK2	Hs01106587_m1
EDN1	Hs01115919_m1
FOXO1	Hs01054576_m1
FRZB	Hs00173503_m1
HSD11B1	Hs01547870_m1
IRS2	Hs00275843_s1
KLF5	Hs00156145_m1
LEP	Hs00174877_m1
MRAP	Hs01588793_m1
PIK3R1	Hs00933163_m1
PPARG	Hs01115513_m1
PPIA	Hs99999904_m1
PTPRQ	Hs01386285_m1
RGS2	Hs01009070_g1
RORA	Hs00536545_m1
SFRP1	Hs00610060_m1
SH3PXD2B	Hs01083721_m1
SOCS1	Hs00705164_s1
SORT1	Hs00361760_m1
TBX15	Hs01070089_m1
VGLL3	Hs01013372_m1
YWHAZ	Hs03044281_g1
ZBTB16	Hs00957433_m1

### Double immunofluorescence microscopy analyses

hBMSCs from the same donor were separately cultured in adipocyte and osteoblast medium on Lab-Tek II CC2 glass chamber slides (Thermo Fisher). At day 14 of differentiation, osteoblasts were incubated in conditioned medium for 48 h (OB-CM) as previously described. As controls, osteoblasts were incubated with serum-free DMEM for the same time (OB). Cells were then fixed with 4% paraformaldehyde for 20 min, and after one wash with Dulbecco’s PBS (DPBS), cells were incubated in immunofluorescence blocking buffer with NH4Cl 50 mM for 5 min. After 3 washes with DPBS, the cells were permeabilized using a 0.2% solution of Triton X-100 in DPBS for 20 min. Primary antibodies against PPARy2 ab45036 (rabbit, 1:100; Abcam) or osteocalcin ab13420 (mouse, 1:100; Abcam) were added to blocking buffer and submitted to overnight incubation at 4°C followed by a 1-hour incubation at room temperature with Alexa 488- or Alexa 568-conjugated goat secondary antibodies (Life Technologies). Fixed and stained cells were mounted for confocal microscopy in SlowFade Gold Antifade reagent with DAPI (Life Technologies). Confocal images were acquired using a Zeiss LSM 780 confocal microscope fitted with an 63X/1.2NA oil immersion objective. The 2012 software package (Carl Zeiss) was used for image capture. The analysis was performed three times (donors D and F). For each experiment, 40 pictures were taken at different areas in the well to form a mosaic (1200 μm x 1000 μm). ImageJ software was used for quantification.

### Methylated DNA quantification assay

The global DNA methylation status was studied by using the Methylflash Methylated DNA Quantification Kit (Epigentek), as previously described [26]. In our experiment, 100 ng of DNA isolated from OB and OB-CC was used for each assay. The absorbance was read using a microplate at 405 nm.

### Immunohistochemical analyses of human bone sections

The immunohistochemical analysis of CD36 was performed on decalcified paraffin-embedded biopsy specimens from elderly women (n = 3, age 50–59 years) and one young woman (n = 1, age 18 years). Iliac crest biopsy specimens from the young women were collected at the anatomy and cytology department (ICO) at Saint-Herblain, France, while iliac crest bone biopsy specimens from elderly women were collected at the endocrinology departments at Odense University Hospital and Aarhus University, Denmark, in accordance with approval by the Danish National Committee on Biomedical Research Ethics, journal nos. S-20070121 and S-20110112. The collecting institutions obtained written, informed consent from all participants. Adjacent 3.5-μm-thick sections were included in the immunostaining. Deparaffinized and rehydrated sections were first antigen retrieved for 20 min at 80°C in citrate buffer (pH 6) and endogenous peroxidase quenched with hydrogen peroxide for 15 min at room temperature. Non-specific antibody binding was blocked using 3% BSA (bovine serum albumin) and 10% normal goat serum in PBS for one hour at room temperature. The sections were incubated with rat anti-human CD36 (1:50, R&D Systems) for one hour at room temperature, incubated with a polymer conjugated with anti-rat antibodies and horseradish peroxidases (Polink-1 HRP, GBI Labs) for 30 min at room temperature, and visualized (brown) using DAB (3,3′-diaminobenzidine, Sigma-Aldrich). Finally, the sections were counterstained with Mayer's haematoxylin. For the negative controls, the primary CD36 antibody was omitted.

## Results

### Nine hours of coculture is sufficient for the conversion of osteoblasts into preadipocytes

**Fig 1:**
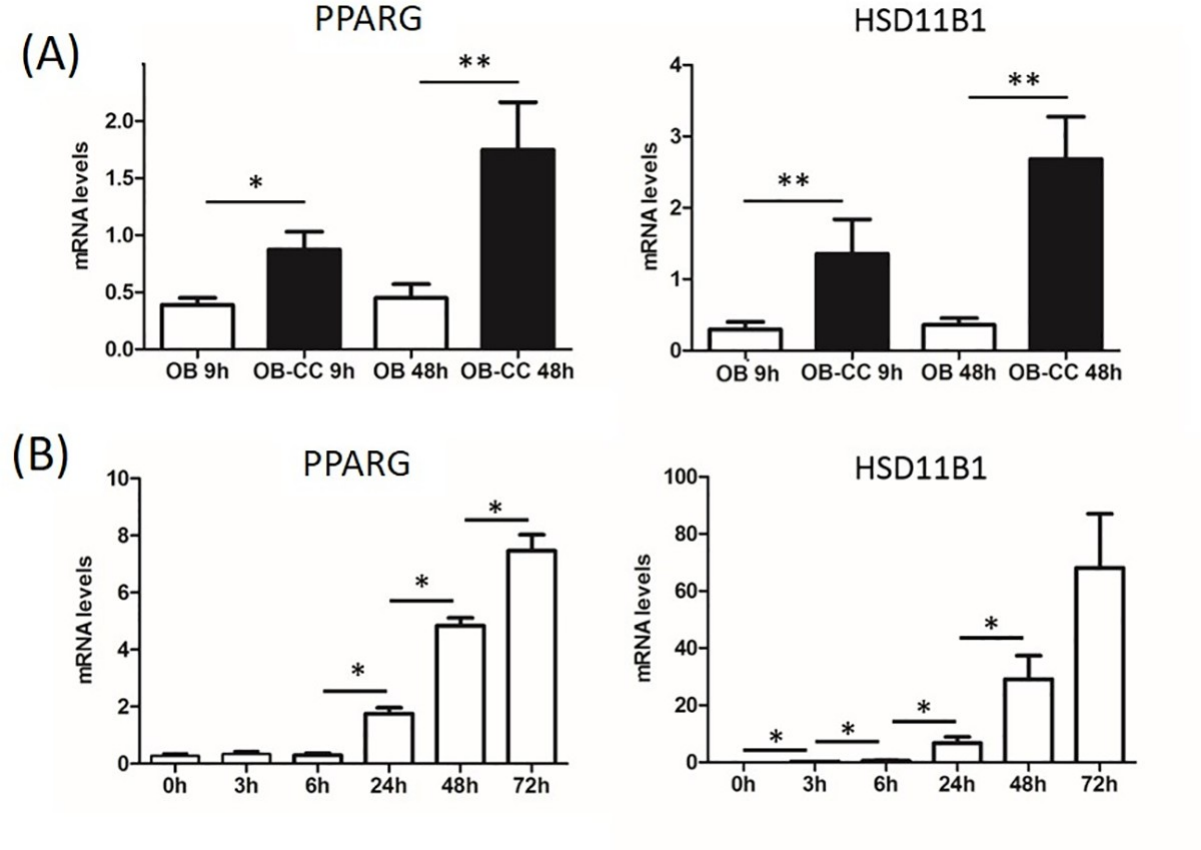
Coculture induced the conversion of osteoblasts into preadipocytes. **(A)** PPARG (Peroxisome Proliferator Activated Receptor Gamma) and HSD11B1 (Hydroxysteroid 11-Beta Dehydrogenase 1) mRNA expression levels in OB and OB-CC after 9 h and 48 h of coculture. mRNA expression levels were normalized against the signal from two housekeeping genes, PPIA (peptidylprolyl isomerase A) and YWHAZ (tyrosine 3-monooxygenase/tryptophan 5-monooxygenase activation protein). Graphs are representative of 6 independent experiments (donors A-F) performed in duplicate. Error bars are the standard deviation. * P ≤0.05, ** P (0.01, cocultured osteoblasts (OB-CC) compared to osteoblasts grown alone (OB). **(B)** PPARG and HSD11B1 mRNA expression levels in BMSC-derived adipocytes at 0, 3, 6, 24, 48, and 72 h of differentiation. mRNA expression levels were normalized against the signal from two housekeeping genes, PPIA and RP2 (RNA polymerase II). Graphs are representative of 4 independent experiments (donors C-F) that were performed in duplicate. Error bars represent the standard deviation. * P ≤0.05.

Gene expression kinetics analyses were performed on cells from six different donors (A-F) to determine the temporal changes in the osteoblast phenotype. The results revealed a time-dependent phenomenon with the first significant increase in mRNA expression levels of adipocyte markers PPARG (Peroxisome Proliferator Activated Receptor Gamma) and HSD11B1 (Hydroxysteroid 11-Beta Dehydrogenase 1) in the osteoblastic population from 9 h of coculture (Fig 1A). In parallel, PPARG and HSD11B1 mRNA levels were determined at various early time points during adipogenic differentiation (donors C-F). This enabled us to show that adipocyte-specific gene expression levels in cocultured osteoblasts (OB-CC) were comparable to those found in adipocytes precociously differentiated (Fig 1B). Consistent with these findings, the expressions of markers representative of mature adipocytes, such as GLUT4 or FABP4, were not detected in OB-CC (data not shown). Taken together, these results indicate that the conversion of osteoblasts is quickly initiated but still incomplete when compared to adipocytes.

### Transcriptomic analysis confirmed the differential gene expression profiles and adipogenic gene induction

**Fig 2:**
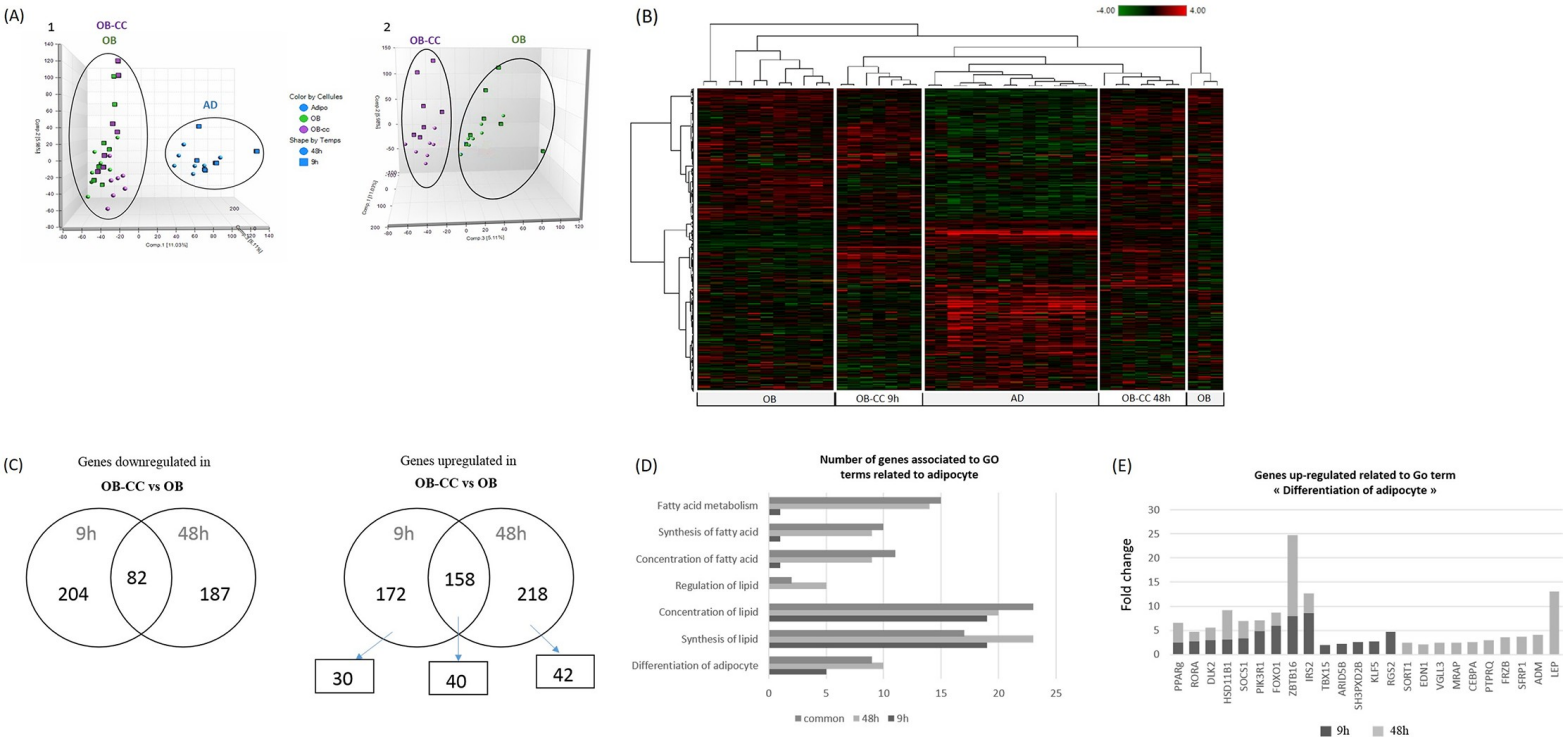
Dynamic transcriptional changes upon coculture. **(A)** Principle component analyses of AD (blue) and OB cells (OB-CC and OB) (1) and OB-CC (pink) and OB (green) (2) using the 30,413 probes that were detected as expressed upon 9 h of coculture (squares) or 48 h (circles). **(B)** Hierarchical clustering of genes that were differentially expressed in AD and OB cells and OB-CC after 9 h and 48 h of coculture. Each row represents an individual gene, and each column represents an individual sample. Relative expression levels are represented as a colour scale from red for lower expressions to green for higher expressions. **(C)** Venn diagram showing the number of genes downregulated and upregulated according to the microarray analyses in OB and OB-CC after 9 and 48 h of culture. Boxes indicate the number of upregulated genes related to adipogenesis. **(D)** Gene ontology (GO) analysis of upregulated genes showed an enrichment of GO terms for biological processes in adipogenesis. The y-axis shows significantly enriched upregulated GO terms in OB-CC versus OB after 9 h and/or 48 h of coculture. The number of upregulated genes for each category is indicated by the ordinate axis. **(E)** Upregulated expression of genes associated with adipocyte differentiation. The y-axis shows the fold change values for differential expression in OB-CC versus OB after 9 and 48 h of coculture.

To further decipher the molecular changes triggered by the coculture, we performed transcriptional analyses using an Agilent 60-mer Sure Print G3 Human Gene Expression Microarray Kit containing 50,599 probes. Transcriptional changes were monitored in 7 biological replicates, each consisting of osteoblasts grown alone (OB) and cocultured osteoblasts (OB-CC) after 9 h or 48 h and adipocytes (AD) at day 14 of differentiation placed in serum-free medium for 9 h or 48 h. Principal component analysis (PCA) was used to delineate variations in the transcriptional data. PCA plots showed 2 large independent clusters along the principal component 1 axis (PCA1) consisting of AD and OB cells (OB and OB-CC). In PC3, we observed that OB and OB-CC samples differed from each other transcriptionally, even if the variations were less prominent (Fig 2A).

To identify significantly differentially expressed transcripts, a general linear model using the LIMMA method and Benjamini-Hochberg false discovery rate multiple testing correction were applied. Comparative analysis of probes with a fold-change cut-off of ≥ 1.3 and a p-value cut-off of ≤ 0.05 revealed that more than 1/3 of the probes were strongly differentially expressed between the 3 kinds of samples. Among them, 2281 and 2391 genes were significantly modified in OB-CC compared with the monoculture controls at 9 h and 48 h, respectively. Hierarchical clustering of differentially expressed genes showed the clear distinction between AD, OB and OB-CC cells (Fig 2B). To gain insight into the robustness of gene expression changes, we selected the genes with a >2-fold increase or decrease in OB-CC versus OB. We identified 172 transcripts that were specifically upregulated and 204 that were specifically downregulated in OB-CC after 9 h of coculture and 218 and 187 transcripts that were specifically upregulated and downregulated after 48 h of coculture, respectively. A total of 158 genes were upregulated, and 82 were downregulated, regardless of the duration of coculture (Fig 2C). Classification of differentially expressed transcripts by Gene Ontology (GO) enrichment revealed that significantly upregulated genes in OB-CC versus OB were particularly enriched for biological processes related to adipogenesis (Fig 2D). In particular, we identified 24 genes that were associated with the differentiation of adipocytes with a fold change (FC) ranging from 2.01 to 16.82. Among them, five genes were only upregulated in OB-CC after 9 h of coculture (2.01 <FC>4.09), and ten genes were upregulated after 48 h of coculture (2.03 <FC>13.02) (Fig 2E). For the genes upregulated in the two conditions, the level of FC gradually increased between the two time points. To assess the transcriptomic data for differential gene expression, quantitative RT-PCR profiles were determined for all the genes associated with the differentiation of adipocytes (Table 3). The results displayed similar levels of variation in expression and confirmed the microarray data analysis.

**Table 3:** Validation of differential expression of genes associated with differentiation of adipocytes using quantitative RT-PCR analysis.

		Transcriptomic analysis results Fold change- OB-CC vs OB		RT-PCR analysis results mRNA levels—OB-CC vs OB	
Gene	Name	9h	48h	9h	48h
PPARG	peroxisome proliferator-activated receptor gamma	2,41	4,20	2,39	3,87
RORA	RAR-related orphan receptor A	2,76	2,00	4,46	1,64
DLK2	delta-like 2 homolog (Drosophila)	2,95	2,67	7,62	4,44
HSD11B1	hydroxysteroid (11-beta) dehydrogenase 1	3,03	6,19	4,86	12,82
SOCS1	suppressor of cytokine signaling 1	3,29	3,64	6,22	3,93
PIK3R1	phosphoinositide-3-kinase, regulatory subunit 1 (alpha)	4,84	2,22	5,05	1,85
FOXO1	forkhead box O1	6,01	2,66	6,18	2,58
ZBTB16	zinc finger and BTB domain containing 16	7,93	16,82	ND	ND
IRS2	insulin receptor substrate 2	8,55	4,13	10,29	1,71
TBX15	T-box 15	2,01	0,00	2,81	1,27
ARID5B	AT rich interactive domain 5B (MRF1-like)	2,15	0,00	1,82	1,39
SH3PXD2B	SH3 and PX domains 2B	2,59	0,00	3,89	1,22
KLF5	Kruppel-like factor 5 (intestinal)	2,68	0,00	1,79	1,19
RGS2	regulator of G-protein signaling 2, 24kDa	4,69	0,00	5,18	1,15
SORT1	sortilin 1	FC<2	2,48	1,85	3,47
EDN1	endothelin 1	FC<2	2,03	2,93	1,75
VGLL3	vestigial like 3 (Drosophila)	FC<2	2,46	1,30	1,19
MRAP	melanocortin 2 receptor accessory protein	FC<2	2,51	1,70	2,36
CEBPA	CCAAT/enhancer binding protein (C/EBP), alpha	FC<2	2,58	0,44	3,60
PTPRQ	protein tyrosine phosphatase, receptor type, Q	FC<2	2,97	0,96	1,70
FRZB	frizzled-related protein	FC<2	3,55	1,20	4,20
SFRP1	secreted frizzled-related protein 1	FC<2	3,67	1,67	4,86
ADM	adrenomedullin	FC<2	4,06	1,92	2,51
LEP	leptin	FC<2	13,02	ND	ND

Fold change (FC) and mRNA expression levels of genes associated with “Differentiation of adipocytes” in OB-CC versus OB after 9 and 48 h of coculture. mRNA expression levels represent the average of 2 independent experiments (donors C and F), which were performed in duplicate and normalized against the signal from the two housekeeping genes PPIA (peptidylprolyl isomerase A) and YWHAZ (tyrosine 3-monooxygenase/tryptophan 5-monooxygenase activation protein). ND: Not determined due to the lack of expression in OB.

### Double immunofluorescence microscopic analyses provide evidence for a transdifferentiation event

**Fig 3:**
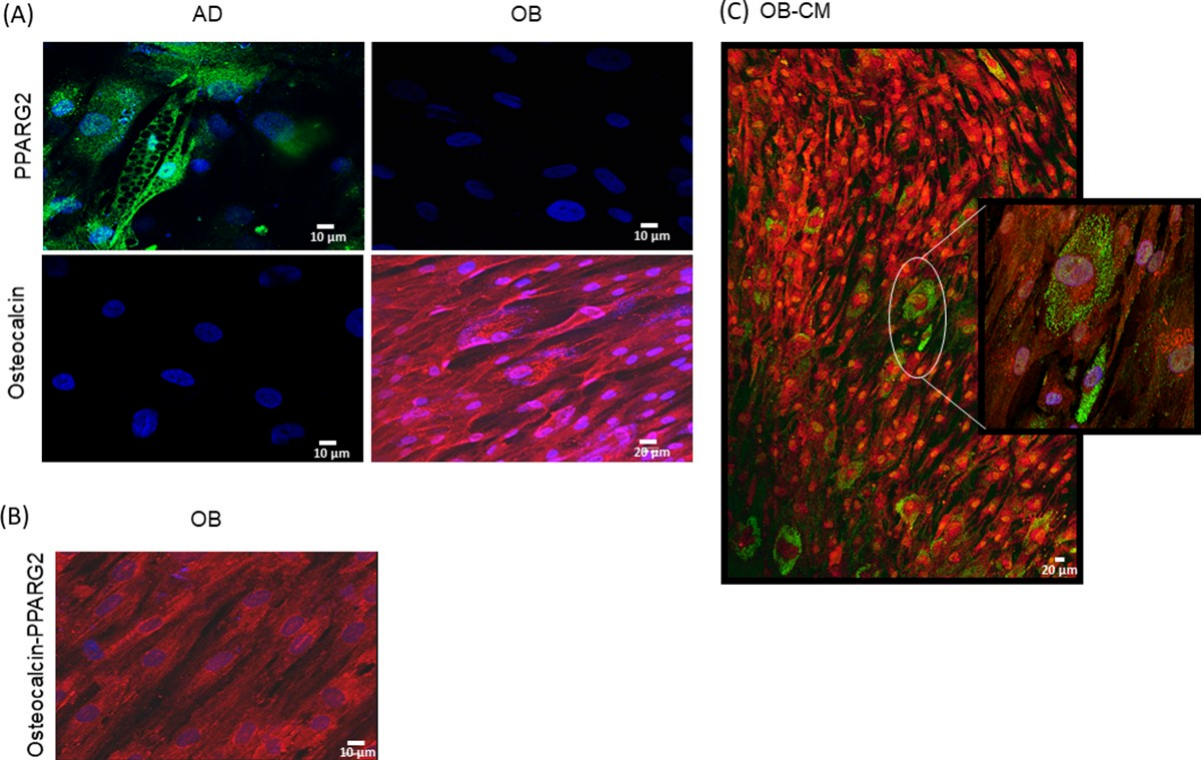
Immunofluorescence microscopic analysis showed the coexpression of adipogenic and osteogenic proteins in OB-CM. **(A)** Simple immunofluorescence confocal analysis showed that osteocalcin is a specific marker for osteoblasts and PPARG2 is a specific marker for adipocytes. **(B)** Double immunofluorescence labelling of PPARG2 and osteocalcin in control osteoblasts showed the specificity of osteocalcin staining in OB but not PPARG2. **(C)** Confocal microscopy analysis of double immunofluorescence labelling of PPARG2 (green) and osteocalcin (red) in osteoblasts incubated with media conditioned by adipocytes at 14 days of differentiation (OB-CM). Blue staining represents DNA (DAPI). The coexpression of the two proteins is clearly visible in the right enlargement. The data shown are representative images of three independent experiments.

Given that BMSCs constitute a heterogeneous cell population, it could be argued that the observed effects of coculture were due to contaminating progenitor cells or undifferentiated cells present in the starting population. Thus, to validate the phenotypic conversion of osteoblasts, we performed double immunofluorescence microscopic analyses with adipogenic and osteoblastic specific markers, peroxisome proliferator activated receptor gamma 2 (PPARG2) and osteocalcin, respectively. Individual cells coexpressing both markers were observed in osteoblasts incubated with media conditioned by adipocytes (OB-CM), providing direct evidence for a transdifferentiation phenomenon (Fig 3). The experiment was performed three times (donors D and F). Image analysis revealed that double-positive cells represented 12.1, 12.1 and 12.8% of the total OB-CM cells, respectively, while no cells showing protein coexpression were observed in the control OB.

### Osteoblast transdifferentiation is associated with increased expression levels of reprogramming genes and with changes in cellular epigenetic status

**Fig 4:**
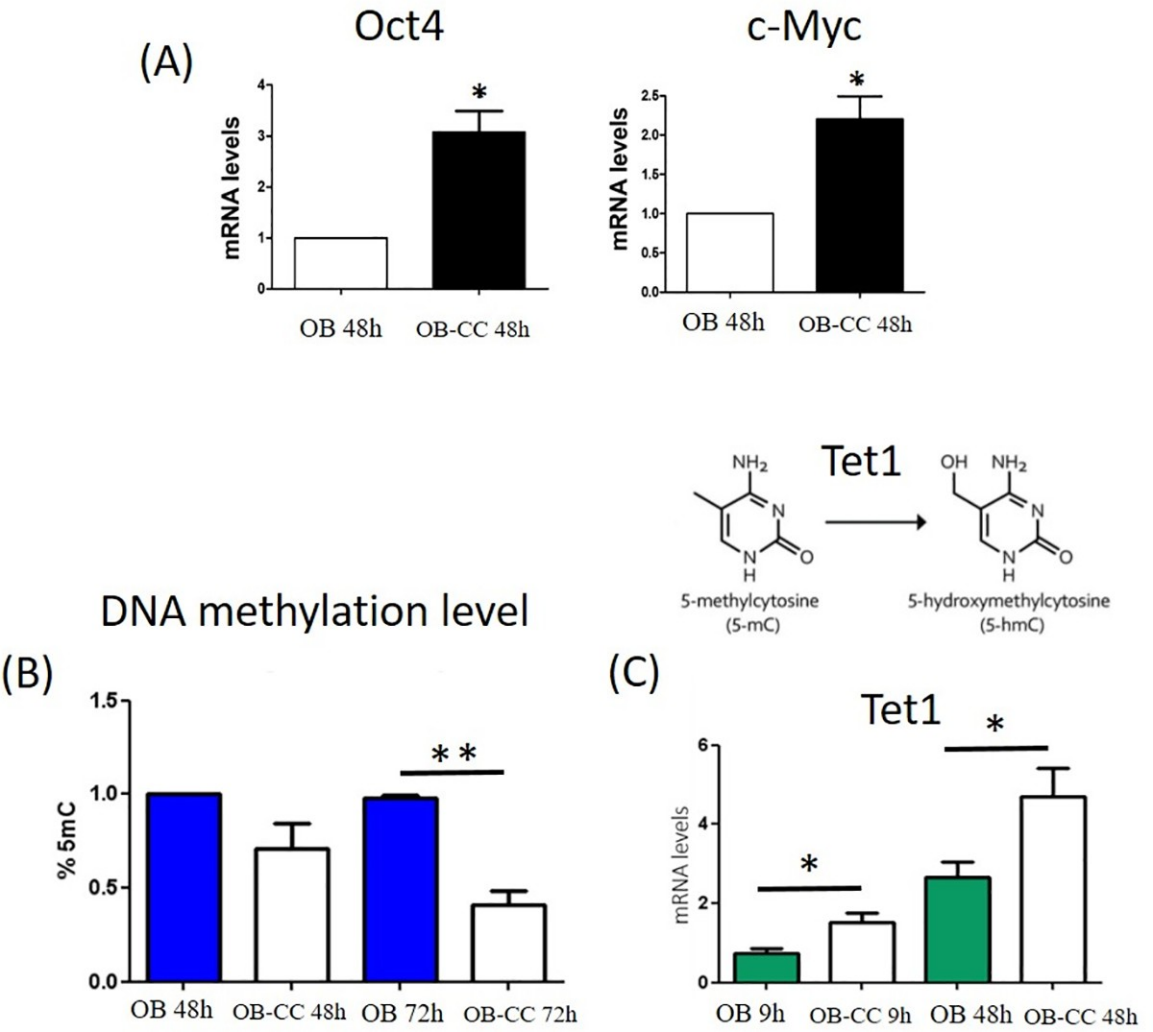
Cell transdifferentiation is associated with a gene-reprogramming event and global epigenetic remodelling. **(A)** OCT4 (octamer-binding transcription factor 4) and c-MYC (oncogene myc) mRNA expression levels in OB and OB-CC after 48 h of coculture. mRNA expression levels were normalized to OB expression against the signal from two housekeeping genes, peptidylprolyl isomerase A (PPIA) and tyrosine 3-monooxygenase/tryptophan 5-monooxygenase activation protein (YWHAZ). Graphs are representative of 4 independent experiments (donors A, C, D, G) performed in duplicate. Error bars show the standard deviation. * P ≤0.05, cocultured osteoblasts (OB-CC) compared to osteoblasts grown alone (OB). **(B)** Percentage of 5-methylcytosine in DNA from OB and OB-CC after 48 and 72 h of coculture. Graphs are representative of 5 independent experiments. Error bars show the standard deviation. No significant changes were observed after 48 hours of coculture, but 72 hours of coculture induced significant hypomethylation. ** P ≤0.01. **(C)** TET1 (ten-eleven translocation methylcytosine deoxygenase 1) mRNA expression levels in OB and OB-CC cells after 9 and 48 h of coculture. mRNA expression levels were normalized to OB expression against the signal from two housekeeping genes for peptidylprolyl isomerase A (PPIA) and tyrosine 3-monooxygenase/tryptophan 5-monooxygenase activation protein (YWHAZ). Graphs are representative of 4 independent experiments performed in duplicate. Error bars show the standard deviation. * P ≤0.05, cocultured osteoblasts (OB-CC) compared to osteoblasts grown alone (OB).

We investigated whether the transdifferentiation event was accompanied by changes in transcripts for genes required for the cell reprogramming process. Quantitative RT-PCR results showed that the mRNA levels of OCT4 (octamer-binding transcription factor 4) and c-MYC (oncogene myc) were significantly upregulated in OB-CC compared to OB (Fig 4A). These data indicate that cell transdifferentiation is associated with a gene reprogramming event, which suggests global epigenetic remodelling. To confirm this hypothesis, the global DNA methylation status was analysed using DNA samples isolated from OB and OB-CC. The results showed that there was a slight decrease in the global DNA methylation level after 48 h of coculture but that this difference between OB and OB-CC cells became statistically significant after 72 h of coculture (Fig 4B). Interestingly, we observed a previous increase in the mRNA expression level of ten-eleven translocation methylcytosine deoxygenase 1 (TET1), the gene encoding an enzyme implicated in DNA demethylation, in cocultured osteoblastic cells versus control cells, consistent with the results at the 5mC level (Fig 4C).

### Functional analysis by gene ontology classification revealed different changes according to coculture duration

**Fig 5:**
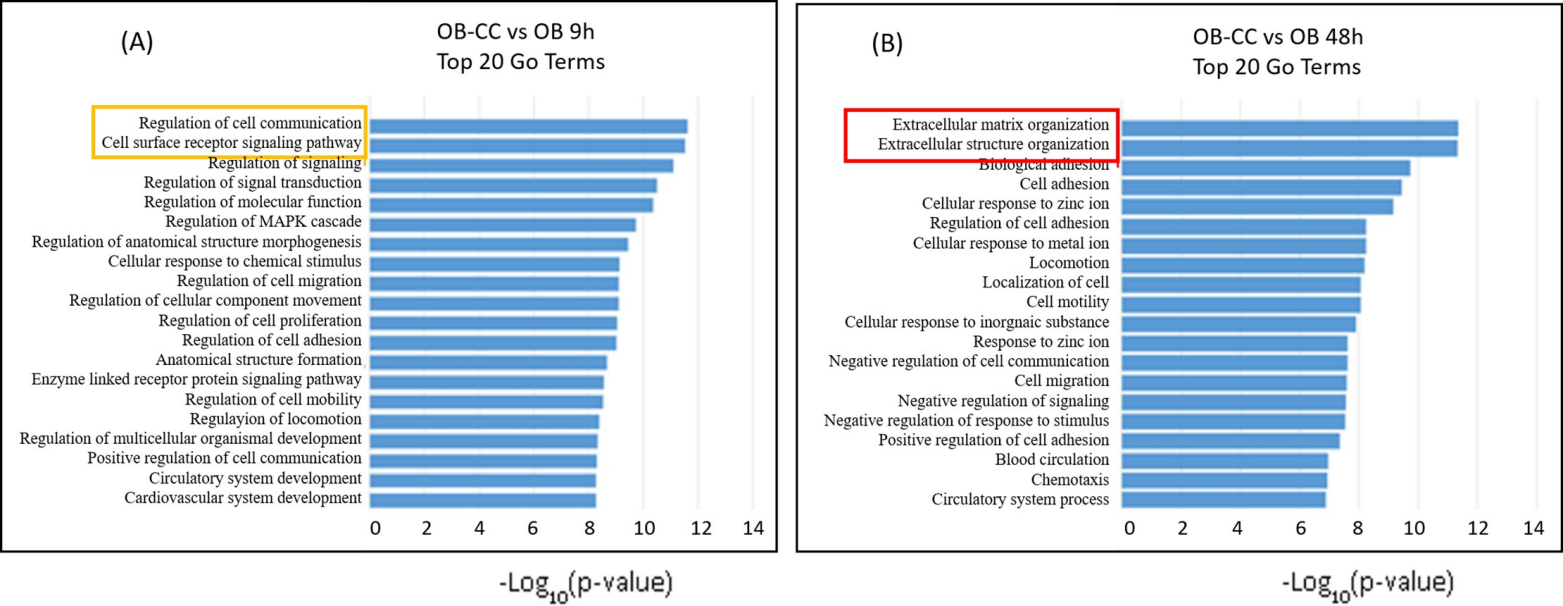
Osteoblasts present different phenotypic changes according to coculture duration. Top 20 GO terms of biological processes (ranked by p-value) for differentially expressed genes found between **(A)** OB-CC 9 h vs. OB and **(B)** OB-CC 48 h vs. OB. Bars represent the minus log_10_ of the p-values.

To understand the mechanisms behind the transdifferentiation event generated by the coculture, differentially expressed probes were subjected to GO analysis. The top 20 GO terms of biological processes revealed that the genes that were significantly up- and down-regulated in OB-CC versus OB upon 9 h of coculture were highly different from those differentially expressed upon 48 h of coculture. Indeed, enriched genes in OB-CC versus OB were first related to regulation of cell communication and cell signalling and then to extracellular matrix organization and cell adhesion (Fig 5). Taken together, these data show that phenotype conversion is initiated rapidly upon coculture and then progresses in a multistep process.

### CD36 immunostaining of human bone

**Fig 6:**
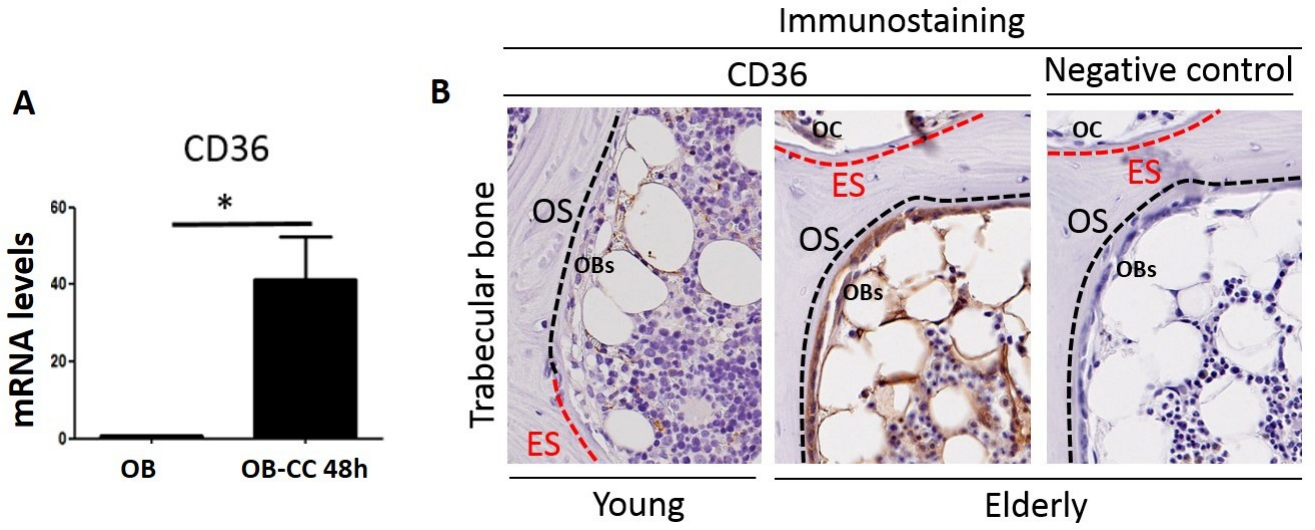
Immunohistochemical analysis confirmed the presence of an adipogenic marker (CD36) in mature osteoblasts in elderly subjects but not in the young subject. **(A)** CD36 mRNA expression levels in OB and OB-CC cells after 48 h of coculture. mRNA expression levels were normalized to OB expression against the signal from two housekeeping genes, peptidylprolyl isomerase A (PPIA) and tyrosine 3-monooxygenase/tryptophan 5-monooxygenase activation protein (YWHAZ). Graphs are representative of 4 independent experiments performed in duplicate. Error bars show the standard deviation. * P ≤0.05, cocultured osteoblasts (OB-CC) compared to osteoblasts grown alone (OB). (B) CD36 immunoreactivity (brown) in human iliac bone biopsy specimens from young and elderly women. Illustrations show the CD36-positive osteoblasts (OBs) on osteoid surfaces (OS) of trabecular bone from an elderly woman and comparable CD36-negative osteoblasts from a young woman. In both the young and elderly women, CD36-positive vascular structures are present, while adipocytes in the marrow cavity appear more CD36-positive in the elderly woman than in the young woman. In the elderly woman, weak staining was also observed in osteoclasts (OC) on eroded surfaces (ES).

To further confirm the presence of osteoblasts that were positive for adipogenic markers, we performed immunohistochemical analysis on human iliac crest bone biopsy specimens from healthy elderly subjects (n = 3) and healthy young subjects (n = 1). CD36 marker, which showed mRNA expression levels that were significantly upregulated in OB-CC compared to OB, was used for immunostaining (Fig 6A).

CD36 was observed in bone marrow adipocytes and vascular structures from all biopsies, as expected. In contrast, CD36 staining was detected in mature bone-forming osteoblasts from both the osteoid surfaces of trabecular bone and in intracortical pores in elderly patients but not in the bone cells of the young patient (Fig 6B). These data constitute a first result that will need to be confirmed with a larger number of subjects.

## Discussion

In humans, progressive infiltration of bone marrow by fat is well established to occur with ageing and supports a causative role for bone marrow adipogenesis in age-related bone loss [7–9]. Osteoblasts and adipocytes are derived from the same multipotent precursor, BMSCs [6, 27], and it has been suggested that the excessive accumulation of marrow adipocytes is caused by preferential differentiation of BMSCs into adipocytes at the expense of osteoblasts [28, 29]. It is also conceivable that the increased adipose tissue may have a negative impact on neighbouring osteoblasts due to its secreted factors, accelerating bone loss and fat accumulation [30]. Supporting this assumption, we previously showed that in the presence of adipocyte-secreted factors, osteoblasts expressed lower amounts of osteogenic markers but exhibited the expression of typical adipogenic genes [20]. In the present study, we more deeply investigated the transcriptional shift of osteoblastic cells upon coculture with adipocytes. Gene expression kinetic and microarray assay data confirmed the quickly initiated adipogenic gene induction and revealed other profound functional changes shortly after the onset of coculture, suggesting cellular conversion. This change in fate is further supported by the panel of experiments described in the paper. Double immunofluorescence staining proved the coexpression of adipogenic and osteoblastic specific markers, excluding the involvement of uncommitted cells in the observed phenomenon. The hybrid phenotype observed in the cocultured osteoblasts, with the expression of both lineage markers, together with the lack of detection of markers representative of mature adipocytes, is indicative of a partial transdifferentiation event [31, 32]. This cellular conversion was preceded by dynamic changes at the transcriptional level, as revealed by the functional annotation of differentially expressed genes (DEGs). With regard to the ‘Biological Process’ group, DEGs were primarily involved in the regulation of cell communication and signalling activity, which is in accordance with the osteoblastic response to an extracellular signal. Then, the enriched DEGs were associated with extracellular matrix (ECM) organization and cell adhesion. As a network of extracellular macromolecules, the ECM interacts with the surrounding cells and modulates functions including cell differentiation, cell adhesion and cell-to-cell communication. Adipocyte lineage commitment is coupled with changes in cell morphology and cytoskeletal components [33]. The modification of ECM components could influence differentiation by permitting cellular rearrangement and changes in cell adhesion properties that are essential for this process [34].

Recent studies have highlighted the importance of epigenetic changes in the lineage determination of adipogenesis and osteogenesis [35, 36]. BMSC differentiation towards a lineage fate is associated with activation of lineage-specific gene expression and repression of other cell type-specific genes [37]. This on-off regulation is partly ensured by promoter DNA methylation. DNA methylation consists of a methyl group covalently bound to the 5 ′position of cytosine in palindromic cytosine-phosphate-guanine (CpG) dinucleotides, which is abbreviated as 5mC. CpG-enriched regions, known as CpG islands, are found in many gene promoters, and their DNA methylation leads to transcriptional repression, while DNA demethylation is linked to transcriptional activation and gene expression. In our model, we found a statistically significant decrease in the global methylation level in osteoblasts upon coculture that is preceded by upregulated expression of TET1, which is one of the three enzymes required for active demethylation. The global demethylation of DNA has been described as a key element in numerous reprogramming events [38]. One of the questions raised by our experiments is why the level of methylation was significantly reduced only after 72 h of coculture, whereas transcriptional changes were detected as early as 9 h. Studies referring to transdifferentiation processes suggest that a triggering effector, such as a transcription factor, microRNA or signalling pathway modulator, could be sufficient to induce lineage conversion upstream of the subsequent chromatin remodelling [31, 32]. One of these effectors could be the OCT4 transcription factor, whose mRNA level was increased upon coculture and which has been described as a potential regulator of TET1 [39], but this remains to be determined.

Unlike cellular reprogramming toward pluripotency, transdifferentiation between closely related cell types can be direct without the involvement of dedifferentiation events but involve a transition through a hybrid intermediate phenotype before acquisition of the phenotypic and functional properties of a mature cell [31, 32]. In our model, we observed a hybrid phenotype in cocultured osteoblasts but without the subsequent conversion into mature adipocytes. This could be explained by the time-limited coculture due to the vulnerability of osteoblasts in a serum-free environment or by the inherent limitations of *in vitro* experiments. Overall, despite these limitations, we are confident in the consistency of our results, which are supported by the panel of experiments conducted on at least 4 donors.

Osteo-adipocyte transdifferentiation related to human bone metabolism has been the focus of several studies [40–42]. The first evidence of *in vitro* transdifferentiation of osteogenic cells was obtained in response to adipogenic agonists [12]. Subsequently, Song and Tuan confirmed the mechanism at the single-cell level by changing the inducing components of the culture medium [11]. In addition to chemical molecules, it has also been shown that physical factors, such as space microgravity, can induce osteo-adipocyte transdifferentiation, even under osteogenic induction conditions [43]. Compared to these studies, the transdifferentiation event observed in our model is not due to an external factor but to the paracrine effect of adipocyte secretions, supporting the hypothesis of the causative role for bone marrow adipogenesis in osteoporotic bone loss.

Of note, although the transdifferentiation concept is still controversial, especially for *in vitro* studies, recent studies have demonstrated that adult cell transition can occur spontaneously *in vivo* under physiological or pathological conditions [44–46]. However, evidence of this mechanism in the physiology of age-related bone loss has not yet been proven *in vivo*. In a preliminary attempt to obtain such clues, we assessed the presence of osteoblasts that are positive for adipogenic markers in human bone biopsies from elderly subjects. We chose to compare them to biopsies of young subjects because communication between osteoblasts and adipocytes is assumed to be controlled by environmental and intrinsic factors, such as cell number, metabolism and secretory profile, which are altered with age [47]. Since the first attempts to label PPARG2 or LPL failed for technical reasons, we used CD36, a cell surface molecule involved in lipid uptake and lipoprotein recruitment for immunostaining [48, 49]. CD36 is expressed mainly in adipocytes, and interestingly, its mRNA expression levels were found to be significantly upregulated in the bone tissue of osteoporotic women compared to controls [50]. In our experiments, we detected the presence of this marker in mature osteoblasts of elderly subjects but not in those of the young subject. Although very promising, these results remain preliminary and will need to be confirmed on a larger number of samples.

In summary, our findings confirm that adipocyte-secreted factors are able to convert osteoblasts into preadipocytes. Further studies are required to elucidate the extracellular signals and the molecular mechanisms and chronological sequence of the conversion. Future work will also focus on consolidating the proof of concept for the role of this transdifferentiation in the pathogenesis of age-related bone loss.
